# Relative Telomere Repeat Mass in Buccal and Leukocyte-Derived DNA

**DOI:** 10.1371/journal.pone.0170765

**Published:** 2017-01-26

**Authors:** Casey T. Finnicum, Conor V. Dolan, Gonneke Willemsen, Zachary M. Weber, Jason L. Petersen, Jeffrey J. Beck, Veryan Codd, Dorret I. Boomsma, Gareth E. Davies, Erik A. Ehli

**Affiliations:** 1 Avera Institute for Human Genetics, Avera McKennan Hospital & University Health Center, Sioux Falls, South Dakota, United States of America; 2 Department of Biological Psychology, Vrije Universiteit, Amsterdam, The Netherlands; 3 Department of Cardiovascular Sciences, University of Leicester, Leicester, United Kingdom; Tulane University Health Sciences Center, UNITED STATES

## Abstract

Telomere length has garnered interest due to the potential role it may play as a biomarker for the cellular aging process. Telomere measurements obtained from blood-derived DNA are often used in epidemiological studies. However, the invasive nature of blood draws severely limits sample collection, particularly with children. Buccal cells are commonly sampled for DNA isolation and thus may present a non-invasive alternative for telomere measurement. Buccal and leukocyte derived DNA obtained from samples collected at the same time period were analyzed for telomere repeat mass (TRM). TRM was measured in buccal-derived DNA samples from individuals for whom previous TRM data from blood samples existed. TRM measurement was performed by qPCR and was normalized to the single copy 36B4 gene relative to a reference DNA sample (K562). Correlations between TRM from blood and buccal DNA were obtained and also between the same blood DNA samples measured in separate laboratories. Using the classical twin design, TRM heritability was estimated (N = 1892, MZ = 1044, DZ = 775). Buccal samples measured for TRM showed a significant correlation with the blood-1 (R = 0.39, p < 0.01) and blood-2 (R = 0.36, p < 0.01) samples. Sex and age effects were observed within the buccal samples as is the norm within blood-derived DNA. The buccal, blood-1, and blood-2 measurements generated heritability estimates of 23.3%, 47.6% and 22.2%, respectively. Buccal derived DNA provides a valid source for the determination of TRM, paving the way for non-invasive projects, such as longitudinal studies in children.

## Introduction

Telomere measurments have been of great interest as a potential tool for assessment of the cellular aging process. The telomere, which caps the end of each strand of DNA, is subjected to attrition throughout the life span due to the end replication problem, which results in the loss of approximately 50–100 base pairs per mitotic division [1]. Once a significant portion of the telomeric region has been lost, the cell enters a state of replicative senescence characterized by a marked change in gene expression, as well as the inability to further divide [2, 3]. Telomere length (TL) has also been implicated in the development of many disorders, either as a marker or causal agent [4, 5].

The telomere region consists of hexanucleotide (TTAGGG) repeat sequences, which are associated with multiple protein factors such as the shelterin complex [6, 7]. Although no exons are contained within the telomeric region, it plays a vital role in genomic protection, stability, and can impact regulation of gene expression elsewhere in the genome [8]. In spite of constant telomere degradation over the life span, mechanisms are available for telomere elongation, mainly through the use of the enzyme telomerase. Telomerase is generally inactive in most somatic cells, but activation is a hallmark of immortal cells [9]. The observation of telomere attrition in proliferating cells, as well as the immortality conveyed via telomerase activation, suggests that telomeres act as a central biological clock mechanism [10]. This is compounded by studies highlighting an association between TL and life span in humans [11–15].

Several studies have addressed the genetic contribution to individual differences in TL in humans [16–19], and specific genomic loci associated with mean leukocyte TL have been identified [16, 20]. In addition to genetic factors, multiple factors such as smoking, sedentary behavior, and periods of high stress, which themselves are partly genetic, may contribute to individual differences in TL [21].

In order to address the role of telomere dynamics in the development of both aging and specific disease pathologies, it is necessary to perform telomere measurements in a longitudinal manner. Investigations into telomere attrition across multiple time points would shed light on differences in telomere attrition over age. However, this presents a challenge as DNA derived from circulating leukocytes obtained by intravenous blood draw is currently the most widely used DNA source for telomere studies. It has been observed that telomere measurments are correlated among somatic tissues regardless of replicative capacities [22–24]. This presents the possibility of utilizing other cellular sources of DNA for telomere measurement studies. Buccal-derived DNA samples are easily collected and are commonly used in biomedical research [25–27]. The use of buccal-derived DNA in place of leukocyte-derived DNA would greatly facilitate large-scale telomere measurement studies. Buccal swab samples are generally composed of buccal epithelial cells, but can also contain a small fraction of leukocytes [28].

The use of qPCR for quantifying relative telomere abundance does not provide an estimate of definitive telomere length due to telomere heterogeneity across chromosomes, rather this technique allows for the determination of the relative abundance of telomere repeat mass present within a sample. Due to this, qPCR based telomere measures are refered to as TRM rather than TL. Here we compared telomere repeat mass (TRM) measures based on buccal-derived DNA with TRM measures based on leukocyte-derived DNA. The blood and buccal samples were obtained in a sample of monozygotic (MZ) and dizygotic (DZ) twins. The twin data provide us with the unique opportunity to estimate the heritability of buccal and blood-based TRM, and to estimate the genetic correlation between the two measures. Our aim is to determine whether TRM measures in buccal-derived DNA are suitable for large scale studies of TRM.

The original leukocyte-derived DNA, which was previously measured for TRM, was subjected to a second TRM measurement to compare the effects of sample handling on TRM measurements. It has been documented that variations in the DNA extraction processes have an impact on telomere measurements, which may have implications for large epidemiological studies [29, 30] as repeated handling of genomic material may lead to changes in the telomere regions; thus altering TRM results. We addressed this issue here as well, as it is relevant to the design of epidemiological studies and to biobanking procedures.

## Methods

### Participants

Blood and buccal samples for DNA extraction were obtained concurrently from individuals in the Netherlands Twin Register (NTR) [31, 32], as previously described [33]. The telomeric DNA from blood samples was measured twice, once in Leicester (England) as a part of a previous study [20] (Blood-1), and once at the Avera Institute for Human Genetics (AIHG; Blood-2). The buccal DNA TRM measurement was performed only once at the AIHG (Buccal). The total sample size comprises 1892 individuals clustered in 1133 families. The individuals include 1809 twins (271 MZ male, 773 MZ female, 156 DZ male, 320 DZ female, and 299 DZ opposite sex), 77 siblings (of whom 12 are multiples; e.g., a member of a triplet), 5 mothers and 1 father. There were 618 MZ twin pairs and 501 DZ twin pairs. Zygosity was based on genome-wide SNP data [34]. The 1892 individuals are distributed over the 1133 families as follows: 1 member (429 families), 2 members (652 families), 3 members (49 families) and 4 members (3 families). Of the 1892 individuals, 589 were males (31%) and 1309 (69%) were females. The mean age was 34.18 years (sd = 13.2, min = 12, max = 78). The Blood-1 TRM measures are available in all 1892 individuals. The measures were distributed over 33 batches (plates), with the mean number per batch equal to 57.3 (sd = 15.3). The Blood-2 TRM measures are available in N = 1338, distributed over 40 batches (mean number per batch 34.3, sd = 12.3). The Buccal TRM measures are available in 1691, distributed over 40 batches (mean number per batch 42.2, sd = 10.5). The study was approved by the Central Ethics Committee on Research Involving Human Subjects of the VU University Medical Center, Amsterdam, NL. All participants and/or parents provided informed consent for the study. Written informed consent was obtained from the approriate next of kin, caretakers, or guardians for all minors present within the study.

## Measurement of Telomere Repeat Mass

The TRM analysis performed in Leicester has been described previously [20]. At AIHG TRM measurements were generated by qPCR to amplify the telomeric region of the DNA, as well as amplification of a single copy gene for normalization purposes. It is important to note that previously generated telomere measurements were referred to as telomere length whereas in this manuscript the results of qPCR based telomere analysis is refered to as telomere repeat mass. The primers used to amplify the telomeric region were tel1B (5’-CGGTTTGTTTGGGTTTGGGTTTGGGTTTGGGTTTGGGTT-3’) (600nM) and tel2B (5’-GGCTTGCCTTACCCTTACCCTTACCCTTACCCTTACCCT-3’) (600nM). The single copy reference gene was amplified using 36b4U (5’-CAGCAAGTGGGAAGGTGTAATCC-3’) (300nM) and 36b4D (5’-CCCATTCTATCATCAACGGGTACAA-3’) (500nM). Each qPCR reaction contained 20 ng of DNA as input, and all samples were run in triplicate (Applied Biosystems ViiA 7). The cycling conditions for both reactions were an initial 95° C for 15 minutes, followed by 30 cycles of 95° C for 15 seconds, and 58° C for 1 minute. Contained within each qPCR batch (telomere and 36b4) was a calibrator sample K562 (Promega, Madison, WI) used for comparison using Cawthon’s calculation method [35]. A standard curve ranging from 100 ng to 6.25 ng was incluced in each telomere and 36B4 qPCR run. TRM was expressed as a ratio of the telomere region (T) to the single copy 36b4 (S), resulting in a T/S ratio. Each TRM measurement was compared to the K562 sample included with each plate, resulting in a relative TRM ratio.

### Statistical Analysis

All TRM measures were first corrected for batch effects by means of an analysis of variance (ANOVA), with batch as the fixed factor. Unless stated otherwise, the residuals were used in the subsequent analyses. We studied the effects of age and sex on TRM and the linear association between the TRM measures with linear regression analysis, using generalized estimating equations (GEE) [36]. We corrected the standard errors for the dependency in the data due to family clustering [37]. We regressed Buccal TRM on Blood-2 TL TRM and on Blood-1 TRM separately. In addition, we regressed Blood-2 TRM on Blood-1 TRM to estimate the association between the blood-based measures of TRM (Table 1). In all analyses, we included sex and age as covariates.

**Table 1:** Regression analyses of TRM measures (standard errors in parentheses).

Dependent	Predictor	b (st err)	% variance
Buccal TRM	Blood-2	0.286 (0.0314)**	10.8% (13.9%)
Buccal TRM	Blood-1	0.208 (0.0183)**	12.2% (15.3%)
Blood-2 TRM	Blood-1	0.373 (0.0180)**	30.4% (39.4%)

** p<0.01;

% variance is the variance explained by the predictor. The percentage in parentheses is due to the predictor + age + sex. The parameter b is the raw regression coefficient in the regression analyses including the covariates age and sex.

The data from twins were used to estimate the contributions of genetic and environmental influences to the phenotypic (co)variance of the TRM measures. Twins raised together form the basis of the classical twin design (CTD), which exploits the fact that MZ twins are genetically (nearly) identical, while DZ twin share on average 50% of their alleles [38–40]. The CTD allows us to fit an ACE model, which includes additive genetic (A), shared environmental (i.e., shared by the twins; C), and unshared environmental effects (E) on TRM. The phenotypic TRM variance is modeled as σ^2^_TRM_ = σ^2^_A_+ σ^2^_C_+ σ^2^_E_, and the twin covariances are modeled as σ_TRM1-TRM2_ = σ^2^_A_ + σ^2^_C_ in MZs, and σ_TRM1-TRM2_ = 0.5*σ^2^_A_+ σ^2^_C_ in DZs. We obtain standardized estimates, usually denoted h^2^, c^2^, and e^2^, by calculating h^2^ = σ^2^_A_/σ^2^_TRM_, c^2^ = σ^2^_C_/σ^2^_TRM_, and e^2^ = σ^2^_E_/σ^2^_TRM_. Note that h^2^ is the heritability (hence h^2^ rather than a^2^, although the notation is arbitrary). Dropping C (or A) from the model reduces the model to an AE (CE) model. The statistical significance of variance components (e.g., σ^2^_C_ or σ^2^_A_) can be tested by means of a likelihood ratio test. It is well established that TRM decreases with age (see Table 2). It is also possible that contributions of A, C, and (or) E to the phenotypic variance changes with age. To investigate this, we fit a moderated ACE model, in which the A, C, and E variance components are free to vary in magnitude with age [41].

**Table 2:** Age and sex effects on batch corrected TRM (standard errors in parentheses).

Variable	N	b (sex)	b (age)	% Variance
Blood-2 TRM	1338	0.0678 (0.0168)**	-0.0067 (0.00067)**	9.0 (7.5)
Buccal TRM	1691	0.0306 (0.0156)*	-0.0036 (0.00059)**	3.1 (2.8)
Blood-1 TRM	1892	0.1130 (0.0217)**	-0.0105 (0.00082)**	10.1 (8.5)

** p<0.01

*p<0.05

% variance is the variance explained by sex and age. The percentage in parentheses is due to age alone. The parameters b are the raw regression coefficients.

The univariate twin model can readily be extended to the multivariate case [42]. That is, we can decompose the phenotypic 3x3 covariance matrix of the 3 TRM measures, **Σ**_TRM_, into genetic and environmental components analogously to the univariate case: **Σ**_TRM_ = **Σ**_A_ +**Σ**_C_ + **Σ**_E_, where the **Σ**_A_, **Σ**_C_ and **Σ**_E_ represent the additive genetic, shared and unshared environmental 3x3 covariance matrices, respectively. The twin 1—twin 2 covariance matrix is modeled **Σ**_TRM1-TRM2_ = **Σ**_A_ + **Σ**_C_ in the MZs, and **Σ**_TRM1-TRM2_ = 0.5*Σ_A_ + **Σ**_C_ in the DZs. This decomposition reveals the contributions of genetic and environmental effects to the phenotypic variances and covariances amongst the TRM measures. We used the full information maximum likelihood (FIML) estimation in the OpenMx R library to fit the twin model models [43]. We first estimated the MZ and DZ covariance matrices. Note that these are 6x6 because we have 3 TRM measures, observed in two twin members. Table 3 contains the FIML estimates of the correlation and covariance matrices in the MZ and DZ twins estimated with age and sex as covariates. Table 3 also includes the number of observed values for each phenotype.

**Table 3:** Full information maximum likelihood (FIML) estimates of twin variances, covariances, and correlations, corrected for sex and age. The correlations are shown below the diagonal in italics. The within phenotypic correlations are underlined. *N* represents the number of observed values.

	**MZ twin 1**			**MZ twin 2**		
	**Blood-1**	**Blood-2**	**Buccal**	**Blood-1**	**Blood-2**	**Buccal**
**N**	375	257	321	390	261	338
Blood-1	0.179	0.062	0.037	0.117	0.057	0.042
Blood-2	*0*.*518*	0.079	0.019	0.055	0.028	0.025
Buccal	*0*.*319*	*0*.*244*	0.076	0.046	0.023	0.036
Blood-1	*0*.*655*	*0*.*466*	*0*.*393*	0.177	0.074	0.044
Blood-2	*0*.*512*	*0*.*384*	*0*.*313*	*0*.*676*	0.068	0.026
Buccal	*0*.*356*	*0*.*320*	*0*.*467*	*0*.*380*	*0*.*354*	0.077
	**DZ twin 1**			**DZ twin 2**		
**N**	522	410	478	522	402	478
Blood-1	0.177	0.060	0.051	0.076	0.043	0.033
Blood-2	*0*.*565*	0.063	0.028	0.032	0.025	0.021
Buccal	*0*.*415*	*0*.*383*	0.086	0.034	0.017	0.030
Blood-1	*0*.*422*	*0*.*296*	*0*.*274*	0.182	0.070	0.038
Blood-2	*0*.*346*	*0*.*345*	*0*.*198*	*0*.*563*	0.085	0.028
Buccal	*0*.*301*	*0*.*311*	*0*.*373*	*0*.*324*	*0*.*356*	0.075

### ACE Modeling

**Fig 1:**
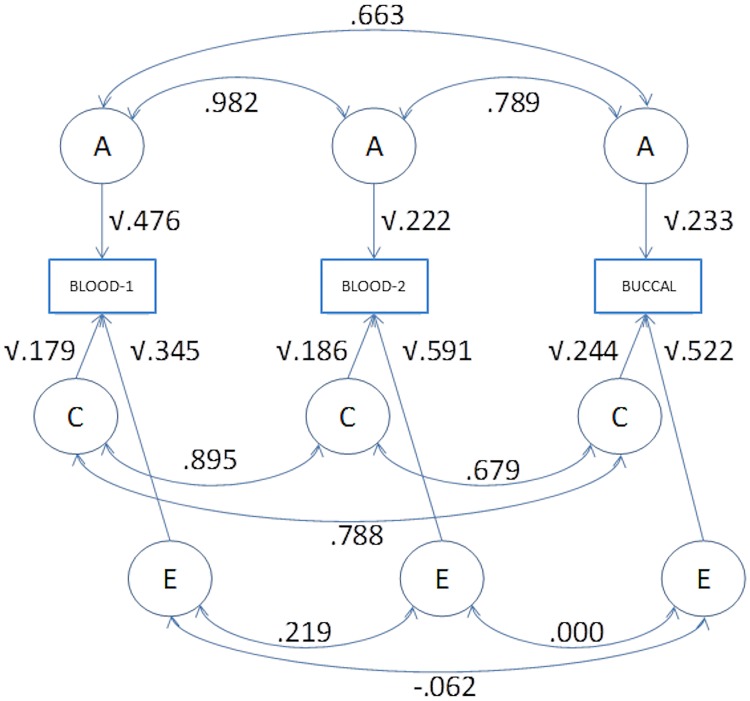
Path diagram depicting the A, C, and E variance components calculated for each of the sample groups.

We investigated the contributions of genetic and environmental influences to the phenotypic covariance matrices by fitting an ACE model, with age and sex as covariates. As mentioned above, in fitting the ACE model, we modeled the 3x3 phenotypic covariance matrix (**Σ**_TRM_)as **Σ**_TRM_ = **Σ**_A_ + **Σ**_C_ + **Σ**_E_, where **Σ**_A_ is the 3x3 additive genetic, **Σ**_C_ is the 3x3 shared environmental, and **Σ**_E_ is the 3x3 unshared environmental covariance matrix. In Fig 1 and in Table 4, we express each covariance matrix as **Σ** = **DRD**^t^, where **D** is a (3x3) diagonal matrix containing the standard deviations, and **R** is the 3x3 correlation matrix. By calculating **Σ**_A_ / **Σ**_TRM_, we obtain the contribution of additive genetic effect to the phenotypic variances (i.e., diagonal elements of **Σ**_A_ / **Σ**_TRM_) and covariances (off-diagonals elements of **Σ**_A_ / **Σ**_TRM_). The diagonal elements are the heritabilities (h^2^). The same applies to the environmental covariance matrices **Σ**_C_/ **Σ**_TRM_ and **Σ**_E_/ **Σ**_TRM_, where the standardized diagonals are the c^2^s and, e^2^s, respectively.

**Table 4:** Parameter estimates in the ACE model. The R_A_ is the additive genetic correlation matrix, and stdev A are the additive genetic standard deviations. Σ_A_/ Σ_TRM_ is the contribution of additive genetic effects to the TRM variances (h^2^) and the covariances (the shared and unshared environmental results are defined analogously). Note that the h^2^, c^2^ and e^2^ estimates appear twice.

	Blood-1	Blood-2	Buccal		Blood-1	Blood-2	Buccal		Blood-1	Blood-2	Buccal
**stdev A**	0.291	0.128	0.135	**stdev C**	0.179	0.117	0.138	**stdev E**	0.248	0.209	0.202
**R**_**A**_	1			**R**_**C**_	1			**R**_**E**_	1		
	0.983	1			0.895	1			0.219	1	
	0.663	0.789	1		0.788	0.678	1		-0.062	0.000	1
**Σ**_A_/ **Σ**_TRM_	0.476			**Σ**_C_/ **Σ**_TRM_	0.179			**Σ**_E_/**Σ**_TRM_	0.345		
	0.549	0.222			0.281	0.186			0.170	0.591	
	0.615	0.554	0.233		0.459	0.447	0.244		-0.074	-0.001	0.522
**h**^**2**^	0.476	0.222	0.233	**c**^**2**^	0.179	0.186	0.244	**e**^**2**^	0.345	0.591	0.522
**CI lower**	0.276	0.070	0.037	**CI lower**	0.013	0.002	0.043	**CI lower**	0.299	0.519	0.456
**CI upper**	0.654	0.437	0.459	**CI upper**	0.363	0.347	0.420	**CI lower**	0.397	0.669	0.595

Finally, we tested whether the estimates of the variance components in univariate ACE models (σ^2^_A_, σ^2^_C_, and σ^2^_E_) varied with age. We did this by modeling σ_A_ as σ_A_ = b_0A_ + b_1A_*age, σ_C_ = b_0C_+b_1C_*age, and σ_E_ = b_0E_ + b_1E_*age [41]. That is, we considered the possibility that the genetic and environmental standard deviations linearly increase (or decrease) with age. The test of age moderation boils down to the test of the omnibus hypothesis: b_1A_ = b_1C_ = b_1E_ = 0.

## Results

Age and sex together explained 9%, 3.1%, and 10.1% of the variance in Blood-2 TRM, Buccal TRM, and Blood-1 TRM, respectively. The percentages of variance explained by age alone equaled 7.5%, 2.8%, and 8.5%, respectively. We tested for the interaction (sex X age), but this interaction was consistently insignificant (p>0.1). Batch effects accounted for 12.4% (TRM Blood-1), 23.1% (TRM Blood-2), and 23.3% (TRM Buccal) of the variance. The zero-order correlations among TRM measures were 0.36 (Blood-2—Buccal), 0.39 (Buccal—Blood-1), and 0.62 (Blood-2 –Blood-1).

### Twin Correlations and ACE Model

Age and sex corrected twin correlations estimated through FIML were 0.655 for MZ twins and 0.422 for DZ twins in the Blood-1 measurements. The Blood-2 measurements performed at the AIHG had MZ and DZ correlations of 0.384 and 0.345, respectively. Similar analyses were performed on the Buccal samples yielding a MZ correlation of 0.467 and a DZ correlation of 0.373 (see Table 5).

**Table 5:** FIML estimates of MZ and DZ twin correlations for the TRM measures (corrected for sex and age), with lower and upper 95% confidence intervals.

	95% lower	correlation	95% upper
MZ Blood-1	0.598	0.655	0.704
MZ Blood-2	0.289	0.384	0.469
MZ Buccal	0.388	0.467	0.537
DZ Blood-1	0.312	0.422	0.516
DZ Blood-2	0.209	0.345	0.464
DZ Buccal	0.268	0.373	0.468

Table 4 contains the parameter estimates of the (trivariate) ACE model, including the estimates of the standardized variance components (h^2^, c^2^, and e^2^), with their upper and lower 95% CIs (last three rows). The heritability of the Blood-1 TRM measure is 0.476, i.e., about 47.6% of the phenotypic variance is due to genetic effects. The C and E effects account for about 17.9% and 34.5% of the TRM variance, respectively. The standardized variance components of the Blood-2 TRM and Buccal TRM equal h^2^ = 0.222 and h^2^ = 0.233, respectively with C effects accounting for 18.6 and 24.4%, and E effects accounting for 51.9 and 45.6% of the Blood-2 and Buccal TRM variance, respectively. We note that the E influences contribute relatively little to the covariance among the TRM measures (see **Σ**_E_/**Σ**_TRM_ in Table 5); whereas both the A and C effects contribute considerably to the covariances among the blood and buccal TRM measures. The genetic correlations between Buccal TRM and the Blood-1 and Blood-2 TRM are 0.663 and 0.789, respectively. The genetic correlation between Blood-1 and 2 TRM is 0.983.

We tested the significance of the A and C effect by dropping these from the model. Dropping C resulted in chi^2^(6) = 10.97 (p = 0.089). In contrast, dropping A resulted in chi^2^(6) = 49.15 (p<0.001). From this, we may conclude that the contributions of C to the phenotypic covariance matrix is not significant, but the contributions of A are. However, the fact that we cannot reject the hypothesis **Σ**_C_ = 0 is likely to be due to a lack of statistical power [44]. We therefore retained the ACE results.

Next, we tested whether variance components were moderated by age. For both blood DNA TRM measures and for buccal TRM measures, the results indicate that there is no evidence to support the hypothesis of age moderation of the variance components σ^2^_A_, σ^2^_C_, and σ^2^_E_, with the chi^2^(3) for Blood-1 being equal to 4.32 (p = 0.23); for Blood-2 1.46 (p = 0.69), and for Buccal 3.38 (p = 0.37). Based on these results we conclude that there is no evidence to support the hypothesis of age moderation of the variance components σ^2^_A_, σ^2^_C_, and σ^2^_E_.

## Discussion

The ability to utilize buccal samples in lieu of a blood sample would greatly increase the ability to perform longitudinal TRM studies. Due to the negligible invasiveness of buccal DNA sampling, future studies may be designed that may span a long period of time; including TRM measurement at birth. Utilizing blood and buccal-derived DNA collected from 1892 participants, mostly twins, we were able to investigate the relationship between DNA derived from different cellular sources, as well as investigate the genetic and environmental components associated with TRM.

The buccal-derived TRM measurements showed a significant association with both sex and age indicating that the TRM data is showing an expected result (telomere attrition). Similar observations have been widely observed in previous studies [45, 46]. This is evidence of similarities in telomere dynamics between the tissue types, which would allow for use of buccal-derived DNA samples for telomere measurement studies. Note that the effect of age on blood based TRM is appreciably greater than the effect on buccal based TRM. This may be due to greater error variance of buccal-derived TRM measurements. We address the contributions of genetic and environmental influences to these phenotypic associations in the analyses of the twin data.

Buccal samples showed a significant phenotypic correlation with both of the blood measurements performed on the same sample multiple years apart. This finding highlights the ability of buccal-derived DNA samples to characterize the cellular aging process in a similar manner as blood-derived DNA. The blood and buccal samples showed similarity compared to measurements regardless of the laboratory performing the assay.

Using twin data to observe phenotypic correlations between MZ and DZ as well as to fit ACE models was informative in yielding estimates of genetic and environmental influences on the TRM phenotype of the sample types under study. Given an AE model we would expect the DZ correlations to be about half the MZ correlations. However, the DZ correlations are clearly larger, which suggests the presence of shared environmental influences [19]. These correlations are consistent with an ACE model. We note that the blood-based TRM measures correlate about 0.58 (0.518 and 0.565 in the MZs and 0.676 and 0.563 in the DZs). The correlation between the blood based and buccal based TRM measures are smaller, ranging from 0.244 to 0.415. The two blood measurements showed a difference in estimated heritability with the Blood-1 estimate at 46.7% of total phenotypic variance, whereas the repeated blood measurement and the buccal sample measurements both showed a heritability estimate of 22.2% and 23.3% respectively. Discrepancies within the TRM measurements replicated on the same blood samples may arise due to a combination of inter-lab variation and possible degradation of samples due to extended handling.

Measurement of the blood samples at different time points allowed for information to be derived concerning the effects of extended handling, as well as inter-lab variation. There have been questions raised regarding the reliability of the relative TRM measurements produced in different laboratories [47, 48]. This study showed a difference in the heritability estimates of TRM both produced in replicated blood samples. The blood samples were first measured in one laboratory, shipped elsewhere, utilized for genomic analysis, and then finally shipped for TRM analysis a second time. It is possible that the extended sample handling, as well as known inter-lab variation in TRM measurement, is responsible for the differences in heritability estimates observed.

Having the ability to easily sample buccal-derived DNA would open the doors to further large scale longitudinal sample collections for TRM measurement. The negligible invasiveness of the collection process makes collection possible from an early age. Cohorts such as those included within the NTR can be followed over multiple time-points in order to investigate temporal effects on TRM throughout an individual’s life span.
